# Potassium channels: Novel targets for tumor diagnosis and chemoresistance

**DOI:** 10.3389/fonc.2022.1074469

**Published:** 2023-01-10

**Authors:** Meizeng Li, Peijie Tian, Qing Zhao, Xialin Ma, Yunxiang Zhang

**Affiliations:** ^1^ School of Basic Medical Science, Weifang Medical University, Weifang, China; ^2^ Department of Pathology, Weifang People’ s Hospital, Weifang, China

**Keywords:** potassium channel, tumor, drug target, chemoresistance, research progress

## Abstract

In recent years, the role of potassium channels in tumors has been intensively studied. Potassium channel proteins are widely involved in various physiological and pathological processes of cells. The expression and dysfunction of potassium channels are closely related to tumor progression. Potassium channel blockers or activators present antitumor effects by directly inhibiting tumor growth or enhancing the potency of classical antitumor agents in combination therapy. This article reviews the mechanisms by which potassium channels contribute to tumor development in various tumors in recent years, introduces the potential of potassium channels as diagnostic targets and therapeutic means for tumors, and provides further ideas for the proper individualized treatment of tumors.

## Introduction

Potassium channels constitute the most prominent family, presenting 77 sequences encoding α pore-forming subunits among the ion channels (1). Potassium channels are the most widely distributed, subtype and complex class of protein molecules found so far, which are commonly involved in the physiological and pathological processes of cells, including cell proliferation and differentiation, pigmentation, migration, cell cycle progression, apoptosis, autophagy, metabolism, angiogenesis, stem cell dynamics and carcinogenesis. Potassium channels also play an essential role in maintaining the function of cells (2). In recent years, the role of potassium channels in cancer cell proliferation, invasion, migration and metastasis has gradually become well-known, suggesting that this channel may become a potential tumor diagnostic marker and therapeutic target. This paper reviews the mechanism of potassium channels in tumor development and introduces its research progress as an emerging candidate target and biological marker for potential anticancer therapy.

## Classification of potassium channels

Potassium channels(K^+^ channels) are the most numerous and diverse ion channels expressed in excitable and non-excitable cells. According to its structure and function, it is mainly divided into four major categories: voltage-gated potassium channels(Kv), calcium-activated potassium channels(KCa), inwardly rectifying potassium channels(Kir), and Two-pore-domain potassium channels(K2P) (3).

## Relationship between potassium channel and tumor

**Figure 1:**
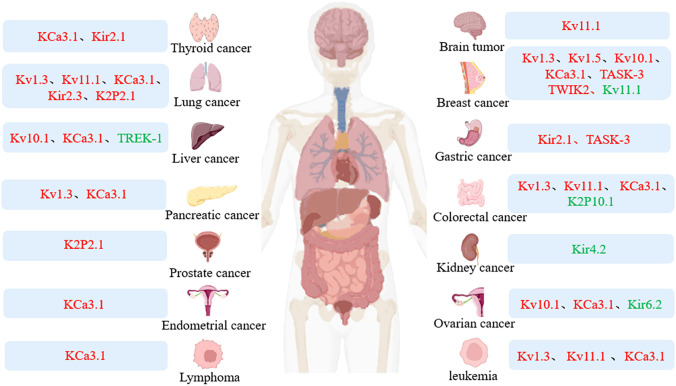
Expression of potassium channel in tumor. Colors represent different expression levels: red, high expression; green, low expression.

Potassium channels are critical regulators of cellular homeostasis and regulate important physiological processes. Intercellular ion redistribution from channel opening can influence cellular function, such as cell proliferation, migration and apoptosis. Abnormal channel expression and function can affect physiological processes and even carcinogenesis. For example, Kv1.3 expression is found to be altered in tissues such as breast cancer, pancreatic cancer, lung cancer, prostate cancer, B-cell lymphoma, and B-cell chronic lymphocytic leukemia(CLL)compared with normal tissues, and its increased activity can promote the development of cancer (1). Therefore, abnormal expression of K^+^ channels is a molecular target of oncogenic transformation, and it is also crucial to explore the relationship between potassium channels and tumors (4) (**Figure 1**).

### voltage-gated potassium channels

Among the different types of K^+^ channels encoded by 77 genes, Kv represents the largest and most complex family (5). There are currently 12 known isoforms(Kv1 – Kv12). They are essential regulators of cellular excitability and signaling and are expressed in most cells (6). Each Kv subunit consists of six transmembrane domains(S1 – S6). Kv is involved in a variety of physiological processes, such as smooth muscle contraction, cell volume control, cell cycle progression, cardiac repolarization and tumor cell proliferation (7). Kv1.3 (8, 9), Kv7.1 (10), Kv10.1 (11) and Kv11.1 (12) plays a vital role in cell cycle regulation and tumor development. Members of the Kv family play essential roles in both cell cycle regulation and apoptosis (13).

Kv1.3(KCNA3), a critical isoform in the Kv family, is selectively permeable to potassium ions and is activated upon changes in membrane potential (14). Overexpression of Kv1.3 channels has been observed in the breast, colon, smooth muscle, skeletal muscle, lymph node, and CLL. However, both Kv1.3 channel expression and Kv1.3 gene promoter methylation can be used as diagnostic and prognostic markers in breast, pancreatic and colorectal cancer(CRC) (9). In addition, the researchers found that Kv1.3 channel blockade was able to lead to phosphorylation of the transcription factor CREB in LUAD A549 cells and play a specific role in cell proliferation, apoptosis and differentiation, thereby affecting the development of lung cancer(LC) cells (15). Kv1.5 overexpression driving resting membrane potential(RMP) hyperpolarization may alter adhesion and morphology of triple-negative breast cancer(TNBC) cells and increase TNBC cell migration and invasion (16).

Kv7 consists of five different ion channels, Kv7.1-7.5, encoded by the KCNQ gene(KCNQ1-5), respectively. Kv7 ion channels, like other Kv channels, are tetramers and require assembly of four α-subunits to produce functional channels (6). KCNQ1 is a tumor suppressor gene in mice, and low Kv7.1 expression has been found to accelerate tumor progression and affect patient survival through analysis of tissue samples from CRC patients (10). There was a positive correlation between Kv7.1-KCNE3 expression levels and survival in CRC patients (17). As an antisense transcript of the KCNQ1 gene, KCNQ1OT1 is up-regulated in BC tissues and acts through the KCNQ1OT1miR-34aNotch3 axis to promote the progression of BC (18). However, the mechanism by which Kv7 channels play a role in tumors and strategies in clinical treatment need to be further investigated.

Kv10.1, also known as ethera-go-go-1(EAG1), is encoded by the KCNH1 gene and plays a critical role in tumor cell proliferation, angiogenesis, migration, and invasion (4). *In vitro* experiments have found that retinoblastoma protein(Rb) can regulate the expression of Kv10.1 and inhibit cancer cell proliferation and tumor development by decreasing the expression level of Kv10.1 (19). Kv10.1 is highly expressed in BC MCF-7 cells. The inhibition of Kv10.1 expression can up-regulate miR-126, thereby reducing the invasiveness of BC cells (20).

Kv11.1, also called ether-à-go-go(HERG), is encoded by the KCNH gene (12). In normal healthy tissues, its expression level is usually low, and it is high in tissues such as leukemia, ovarian cancer(OC), LC, and BC (21). Activation of Kv11.1 promotes Cav-1 dephosphorylation thereby reducing BC MDA-MB-231 cell migration and invasion (22). In glioblastoma(GBM) xenografts, high expression of HERG may be associated with the proliferation (23). In CRC cell lines, HERG1 presents high expression, and knockdown of HERG1 reduces the proliferation and tumorigenic ability of CRC cells (24). HERG1 is similarly overexpressed in hepatocellular carcinoma(HCC) but restricted to the early stages of tumor development (25).

### calcium-activated potassium channels

Calcium-activated potassium channels are divided into large conductance calcium-activated potassium channels(KCa1.1), medium conductance calcium-activated potassium channels(KCa3.1), and small conductance calcium-activated potassium channels(KCa2.1-2.3). The large conductance potassium channel(BK) is a tetramer composed of six transmembrane domains(S1 – S6), and the central pore of each transmembrane domain is located in the S5 – S6 region. Small versus medium conductance(SK) is a tetramer composed of a and b subunits, in which the pore is formed by one subunit (26). KCa channels are expressed in almost all human cells and are essential determinants of hyperpolarization following action potentials and are widely involved in cellular activities such as cell cycle, proliferation, migration, and apoptosis.

KCa3.1 channels, also known as SK4 or IK, are encoded by the KCNN4 gene and are activated by elevated intracellular Ca^2+^ concentrations (27). KCa3.1 is a common oncogene. It has been found that KCa3.1 is involved in the development of OC, and blocking or down-regulating KCa3.1 expression in OC cells can reduce the migration of OC (28). KCa3.1 is highly expressed in TNBC and can promote migration and epithelial-to-mesenchymal transition(EMT) of TNBC cells (29). KCa3.1 is involved in cell proliferation by regulating G1/S transition and is a critical regulator of cell cycle progression and proliferation in BC (30). In endometrial cancer(ECa), the expression level of KCa3.1 is significantly upregulated in EC tissues compared with normal tissues, and lncRNA-14327.1 regulates migration and invasion of EC cells by stabilizing KCa3.1 protein and activating EMT (31). In HCC, KCa3.1 promotes invasion and metastasis *in vitro* by inducing MAPK/ERK and EMT pathways. Down-regulation of KCa3.1 can inhibit the proliferation ability of HCC cells (32). Another study similarly found that KCa3.1 promotes cell cycle progression and promotes cell migration and invasion in HCC by activating protein kinase 2(SKP2) and inducing EMT (33). KCa1.1 channel blockers encoded by KCNMA1 did not significantly alter the viability of HCC Huh7 cells under normoxic conditions but reduced cell proliferation capacity under hypoxic conditions. *In vivo* experiments revealed that blocking KCa1.1 channels inhibited hepatoma cell migration and invasion (34).

### Inwardly rectifying potassium channels

The inward rectifier potassium channel family is a class of non-voltage-gated K^+^ channels, and current data suggest that there are seven subfamilies of Kir channels(Kir1.1 to Kir7.1), which can be usually divided into four categories according to function: classical Kir channels(Kir2.X), G-protein-activated Kir channels(Kir3.X), ATP-sensitive potassium channels(Kir6.X), and K + transport channels(Kir1.X, Kir4.X, Kir5.X, and Kir7.X) (35, 36). Kir is widely distributed in various tissues of the human body and has a variety of physiological functions, playing a pivotal role in maintaining resting potential, controlling cell excitability, and regulating cell volume, and its activity is controlled by a variety of mediators, such as binding proteins, phospholipids, and ions (37).

Kir2.1(KCNJ2) is associated with the development of papillary thyroid carcinoma(PTC), and interference with KCNJ2 inhibits cell proliferation, invasion, migration, and EMT processes (38). The researchers found that the lncRNA POU6F2-AS1 may act as a competitive endogenous RNA(ceRNA) for miR-34c-5p and promote the expression of Kir2.3(KCNJ4) and enhance the invasiveness of LC cells (39). Kir6.2(KCNJ11) and Sulfonylurea Receptor(ABCC9) gene expression is downregulated in OC compared to healthy tissues, and expression of Kir6.2 correlates with OC prognosis (40). In addition, Kir4.1(KCNJ10) expression is low in astrocytomas and oligodendrogliomas. MiR-5096, as a potential target of the KCNJ10 gene, can decrease Kir4.1 channel protein expression in GBM cells, and miR-5096 and Kir4.1 knockdown can increase glioma cell invasiveness (41). Kir4.2(KCNJ15) is lowly expressed in renal cell carcinoma(RCC). KCNJ15 overexpression inhibits RCC cell proliferation, migration, inhibits cell cycle and induces apoptosis by affecting EMT and matrix metalloproteinase-7 and p21 expression (42).

### Two-pore-domain potassium channels

Two-pore domain potassium channels are a class of ion channels encoded by the KCNK gene family and have a unique membrane topology of four transmembrane helices and two pore domains. Based on their unique primary structure, physiological properties, and biological, the K2P channel family has 15 members divided into six subgroups(THIK, TASK, TRESK, TWIK, TALK, and TREK) functions. K2P channels are widely distributed and are involved in regulating cellular function and maintaining resting membrane potential (43, 44). Abnormal expression and dysfunction of K2P channels are closely related to the development of cancer (45).

TREK-1(KCNK2), a member of the K2P family, is by far the most studied K2P channel, and KCNK2 expression is decreased in HCC (46). Researchers have found that TASK-1(KCNK3) inhibits LUAD cell proliferation and glucose metabolism by regulating AMPK-TXNIP signaling pathway (47). In non-small cell lung cancer(NSCLC) cell line A549, TASK-1 presented high expression and was able to promote cell proliferation as well as inhibit apoptosis (48). Down-regulated lncRNA KCNK15-AS1 in pancreatic cancer(PC) tissues inhibits PC BxPC-3 cell invasion (49). TWIK-2(KCNK6) expression is significantly increased in BC, and its overexpression enhances the proliferation, invasion and migration of BC cells and may be involved in the malignant transformation of BC (50). TASK-3(KCNK9) promotes the proliferation and survival of cancer cells by enhancing their resistance to hypoxia and serum deprivation. Knockdown of TASK-3 in BC MDA-MB-231 cells reduced proliferation while inducing cellular senescence and cell cycle arrest (51). In gastric cancer(GC) cell lines, TASK-3 is a crucial protein involved in migration and cell survival, and low TASK-3 expression decreases cell proliferation and migration (52). In addition, TASK-3 was significantly upregulated in oral squamous cell carcinoma tissues in a rat model, and both TASK-1 and TRESK were downregulated in advanced poorly differentiated oral squamous cell carcinoma (53). KCNK15 is highly expressed in PC cells, and researchers have found that KCNK15-AS1 hinders the migration and proliferation of PC cells by regulating KCNK15 and the tumor suppressor gene PTEN (54).

## Role of potassium channel in diagnosis and treatment of tumor

Potassium channels play a crucial role in cancer biology as one of the most widely distributed ion channels, the expression and dysfunction of potassium channels are closely related to tumor progression (**Table 1**). Potassium channels are aberrantly expressed in many tumor cells and play an essential role in cell proliferation and differentiation. Many studies have shown that pharmacological inhibition of specific isoforms is an effective method to inhibit proliferation, migration and invasion, as well as to increase apoptosis (65). Potassium channels can not only be used as markers for tumor diagnosis and prognosis but also provide new ideas for targeted cancer therapy.

**Table 1:** Mechanism of potassium channel involved in tumor development.

Potassium ion channel	Cancer types	Expression	Regulatory molecules	Functional role	References
Kv1.3	Lung cancer	overexpression	CREB phosphorylation	Proliferation、Apoptosis	(15)
	acute lymphoblastic leukemia	overexpression	AKT/ERK1/2/MYC signaling pathways	Drug target	(55)
	chronic lymphocytic leukemia	overexpression	n.d.	Apoptosis	(56)
	leukemia	overexpression	n.d.	Apoptosis、Drug target	(57)
	Pancreatic cancer	overexpression	n.d.	Drug target	(58)
Kv1.5	Breast cancer	overexpression	Cadherin-11/MAPK pathway	Migration	(16)
Kv7	Colorectal cancer	downregulation	n.d.	Tumor inhibition	(10, 17)
Kv10.1	Retinoblastoma	overexpression	n.d.	Promotion of tumor progression	(19)
	Breast cancer	overexpression	MiR-126	Tumor inhibition	(20)
	Acute myeloid leukemia	overexpression	n.d.	Drug target、Poor prognosis	(59)
Kv11.1	Breast cancer	downregulation	Facilitates Cav-1 dephosphorylation	Reduced cell invasion	(22)
	Glioblastoma	overexpression	n.d.	Proliferation	(23)
	Medulloblastoma	overexpression	n.d.	Drug target	(60)
KCa1.1	Liver cancer	overexpression	n.d.	migration	(34)
	Cervix carcinoma	overexpression	n.d.	Diagnostic markers	(61)
KCa3.1	Ovarian cancer	overexpression	P2y2 Purinergic	migration	(28)
	Breast cancer	overexpression	EMT pathways	Proliferation 、migration	(29)
	Liver cancer	overexpression	MAPK/ERK/EMT pathways	Proliferation	(32)
			SKP2/EMT pathways	Migration	(33)
	Papillary thyroid carcinoma	overexpression	EMT pathways	Migration	(38)
	Pancreatic cancer	overexpression	n.d.	Poor prognosis	(62)
	Leukaemia	overexpression	n.d.	Drug target	(63)
Kir2.1	Thyroid cancer	overexpression	EMT pathways	Proliferation 、migration、Poor prognosis	(28)
	gastric cancer	overexpression	Serine/Threonine protein kinase 38	prognostic marker	(64)
Kir2.3	Lung cancer	overexpression	lncRNA POU6F2-AS1/miR-34c-5p	Poor prognosis	(39)
Kir4.2	Kidney cancer	downregulation	EMT pathways/Matrix metalloproteinase-7/P21	Proliferation、migration、Apoptosis	(42)
Kir6.2	Ovarian cancer	downregulation	n.d.	Prognosis	(40)
TREK-1	Liver cancer	downregulation	n.d.	Prognosis	(46)
TASK-1	Lung cancer	overexpression	AMPK-TXNIP Pathway	Proliferation、migration、Apoptosis	(47)
TASK-3	Breast cancer	overexpression	CDK/Cyclin complexes	Proliferation	(51)
	Gastric cancer	overexpression	n.d.	Proliferation、Apoptosis	(52)
TWIK-2	Breast cancer	overexpression	n.d.	Proliferation、Apoptosis	(50)

### Potassium channels: diagnostic markers and drug targets for tumors

Kv1.3 is located in the plasma membrane and intracellularly and plays a vital role in cell proliferation and apoptosis (66). The specific Kv1.3 channel inhibitor clofazimine, as a membrane-permeable small molecule organic compound, can inhibit cancer cell proliferation and induce cancer cell apoptosis through the mitochondrial pathway (9). The researchers found that the Kv1.3 inhibitor tetrahydropyran inhibited PC cell proliferation and induced apoptosis (58). *In vitro* experiments revealed that the mitochondrial Kv1.3 inhibitors PAPTP and PCARBTP were able to promote apoptosis in multiple myeloma cell lines L-363 and RPMI-8226 (67). Mitochondrial targeting inhibitors have been found to alter mitochondrial function by inhibiting Kv1.3 leading to reactive oxygen species mediated apoptosis of cancer cells *in vivo in vitro* and *in vivo* experiments in melanoma and pancreatic ductal adenocarcinoma (PDAC) (68). The Kv1.3 channel inhibitor memantine promoted acute lymphoblastic leukemia(ALL) cell death through AKT, ERK1/2, and MYC signaling pathways. Combined with AraC caused ALL cell proliferation arrest and cell death by increasing CytC release as well as promoting caspase-9 and caspase-3 activation (55). Kv1.3 channels are the only voltage-dependent potassium channels in the plasma membrane of human lymphocytes (69).Compared with healthy lymphocytes, Kv1.3 is highly expressed in the plasma membrane and mitochondria of human CLL cells. The mitogenic agent PAPTP, which specifically targets this channel, effectively kills pathological B cells in the spleen and peritoneal cavity. While inducing apoptosis in CLL B cells expressing high levels of Kv1.3, PAPTP does not alter the survival of healthy B and T cells (56). PAP-1, Psora-4, and clofazimine are inhibitors of Kv1.3 channels that induce apoptosis in human Jurkat leukemia T cells by inhibiting intracellular Kv1.3. They can also induce Bax/Bak-deficient human Jurkat leukemia T cell death. Studies have shown that Kv1.3 inhibitors are potent inducers of apoptosis in mitoKv1.3-expressing tumor cells and their mode of action is independent of Bax and Bak, which provides a new strategy for finding resistance mechanisms in tumor cells as well as identifying new targets for chemotherapy (57).

Kv10.1 is a standard tumor marker that is overexpressed in approximately 70% of human tumors and cancer cell lines, and channel inhibition reduces tumor growth (70). Chloroquine, as a Kv10.1 channel inhibitor, can inhibit outward potassium currents in BC cells, thereby reducing cell migration (71). Proanthocyanidin B1, a natural compound extracted from grape seeds, is a specific inhibitor of Kv10.1 channels, and inhibition of Kv10.1 currents can inhibit migration, proliferation, and xenograft tumor development in the hepatoma cell line Hh-7 cells as well as HepG2 cells (72). The researchers found that fusion of a single-domain antibody(nanobody) to Kv10.1 with tumor necrosis factor-related apoptosis-inducing ligand TRAIL showed strong apoptosis-inducing effects in different tumor models (73). The expression level of hEag1 is closely associated with shorter expected survival in acute myeloid leukemia(AML)patients. In AML, the authors found an interesting isoform-dependent phenomenon of hEag1 expression, with half of the cases of the most common subtypes M2 and M4 expressing hEag1, which is associated with increased age, higher relapse rates, and significantly shorter overall survival. However, hEag1 expression was not detected in CLL. The hEag1 blocker astemizole can increase the apoptotic response, inhibit cell migration in AML cells, and may serve as a potential target for treating AML (59).

Kv11.1 activator NS1643 exerts antitumor effects on TNBC *in vivo* and *in vitro* by causing DNA damage *via* a Ca^2+^ dependent mechanism (74). High Kv11.1 expression is associated with a favorable prognosis in estrogen receptor-negative breast cancer, and NS1643, a Kv11.1 activator, reduces the metastatic spread of breast tumors *in vivo* by inhibiting cell motility *via* β-catenin (75). Researchers have found that Kv11.1 is highly expressed in precancerous lesions of the gastrointestinal tract, and Kv11.1 can be used as an early diagnostic marker for gastrointestinal tumors (76). In addition, Kv11.1 is widely expressed in metastatic CRC, and combined treatment with Kv11.1 blockers and bevacizumab inhibits local tumor growth and metastatic spread (77). Another *in vitro* and *in vivo* experiment on the CRC model, clarithromycin, an antibiotic targeting Kv11.1, induced apoptosis and increased the cytotoxic effect of 5-fluorouracil (78). In addition, the researchers found that KCNQ1 can be used as a robust prognostic indicator of disease recurrence in patients with stage II and III colon cancer (79). Medulloblastoma(MB) is children’s most common malignant brain tumor, and EAG2 can promote brain tumor growth. Antipsychotic Thioridazine, an EAG2 channel blocker, reduces xenograft MB growth and metastasis. Analysis of case reports in patients receiving thioridazine showed a reduction in the extent of disease following thioridazine treatment. However, HERG channel blockers are at risk for cardiac arrhythmias, tardive dyskinesia, and ataxia, and it is essential to monitor closely for possible side effects (60). Dichloroacetate(DCA) is a metabolic modulator widely used in the treatment of inherited mitochondrial diseases. It has been found that DCA can activate Kv channels in all tumor cells, inhibit tumor growth, induce apoptosis by inhibiting mitochondrial pyruvate dehydrogenase kinase(PDK) and converting cell metabolism from glycolysis to aerobic oxidation of glucose, and this effect does not occur on normal cells (80). A large number of peptide toxins contained in scorpion venom act by regulating Kv activity and affect the activity of cancer cells, thus exerting anti-tumor effects (81). Thus, these studies suggest that effective strategies to use drugs that target ion channels for cancer treatment are beneficial.

KCa1.1 channel is a potential early marker of human cervical cancer(CC), and human cervical biopsies show differential KCNMA1 protein expression, and detection of this channel in CC screening programs may be helpful for early detection of CC (61). KCa2.2(KCNN2) is strongly associated with melanoma. Miconazole, a known cytochrome P-450 inhibitor, showed highly high antiproliferative activity in KCNN2-mediated melanoma cell lines (82). The KCNN3 gene encodes KCa2.3. Compared with normal, the expression levels of KCNN3 mRNA and protein were significantly lower in OC tissues, and low KCNN3 expression was associated with poor prognosis in OC (83). Alternatively, SigmaR1 acts as a stress-activated partner and requires increased calcium influx by triggering KCNN3. This drives BC and CRC cell migration and promotes tumor development. High SigmaR1 expression is associated with reduced overall survival in BC (84).

KCNN4 is highly expressed in PDAC. The combination of KCNN4 with TNM stage, lymph node metastasis and histological differentiation is an independent prognostic factor for overall survival in patients with PDAC (62). Increased KCa3.1 expression correlates with invasion of NSCLC cells. KCNN4 DNA hypomethylation and KCa3.1 overexpression have been found to correlate with poor prognosis in NSCLC and are also strong independent predictors of survival in patients with NSCLC (85). KCNN4 promotes the progression of PTC by inducing EMT and inhibiting apoptosis, and its expression correlates with disease-free survival, immune infiltration, and several other clinicopathological features. It can be used as a diagnostic and prognostic biomarker for PTC (86). In glioma cells, temozolomide(TMZ) exerts antitumor effects by inhibiting KCNN4 channel activity (87). The prototypical leukemic T-cell line Jurkat showed lower Kv1.3 and KCa current densities compared to healthy T-cells and other T-cell lines. In activated T cells upregulated by KCa3.1, Ca^2+^ influx is reduced after the application of KCa3.1-specific blockers, and selective KCa3.1 blockers may become a valuable view for anti-leukemia therapy (63). Another study found that ionizing radiation used clinically for fractionated radiotherapy doses was able to activate K^+^ channels, resulting in increased membrane conductance, inhibition of cell proliferation and migration, and induction of apoptosis in LUAD cells (88). Small molecule and channel-targeting antibodies can be used for imaging KCa3.1 channels *in vitro* and *in vivo*. Because KCa3.1 channel expression has predictive potential for prognosis and patient survival in different tumor entities, imaging probes targeting KCa3.1 channel expression can serve as necessary diagnostic tools (89).

ATP-sensitive potassium channel(KATP) is an inwardly rectifying potassium channel. Minoxidil acts as an activator of KATP channels and decreases TNBC cell invasion in combination with ranolazine (90). Kir2.1 is an important regulator of invasion of human GC. The oncogenic effect of this channel depends on its interaction with serine/threonine protein kinase 38(Stk38). Kir2.1 is highly expressed in GC and is positively correlated with depth of tumor invasion, metastatic status, and poor overall patient survival, and is a potential prognostic marker and therapeutic target (64). Kir2.3 overexpression is strongly associated with poor prognosis in patients with LUAD (39). In OC, minoxidil prevents tumor growth in OC xenograft models by stimulating Kir6.2/SUR2 channels to produce mitochondrial destruction and extensive DNA damage, altering the metabolic and oxidative status of cancer cells (40).

BL1249, a K2P2.1(TREK1) activator, was able to inhibit the proliferation and migration of the human PDAC cell line BxPC-3 cells (91). Several other subfamily members of K2P function to sense or switch mechanical stimuli and can serve as tumor diagnostic markers. K2P2.1 expression is elevated in LC but decreased in breast, gastrointestinal, and head and neck cancers. K2P2.1 is expressed in prostate cancer(PCa) but not found in normal prostate epithelial cells. K2P10.1 is down-regulated in CRC and renal clear cell carcinoma (92). In addition, in HCC, the expression of KCNK2, KCNK15, and KCNK17 is decreased, and KCNK9 expression is increased, which are all associated with a good prognosis in patients with liver cancer (46). In TNBC, high KCNK5, KCNK9, and KCNK2 expression showed poor prognosis (45). Alternatively, in thyroid cancer(TC) tissues, KCNK2, KCNK4, KCNK5, and KCNK15 play regulatory roles in the carcinogenesis and metastasis of TC and may serve as a potential therapeutic target in the future (93). Therefore, K^+^ channels can serve as a potential tumor-specific drug target, and targeting K^+^ channels alone or in combination with chemotherapy may become a promising new strategy for anticancer therapy (94).

### Role of potassium channel in chemoresistance of tumor

In cancer therapy, multidrug resistance has been a major clinical barrier to cancer therapy, and 90% of chemotherapy failure cases are associated with tumor resistance (95). Kv10.1 is involved in the chemoresistance of OC cells. Decreased Kv10.1 expression is associated with good prognosis by tissue samples from patients treated with cisplatin chemotherapy and may serve as a potential indicator for predicting chemosensitivity (96). Cells endogenously overexpressing Kv10.1 were also more sensitive to inhibitors of mitochondrial metabolism than cells with low expression. Inhibition of Kv10.1 expression or function results in mitochondrial fragmentation, increased reactive oxygen species, and increased autophagy, providing novel strategies to overcome drug resistance in cancers with high Kv10.1 expression (97). In an antibody-based treatment strategy targeting Kv10.1, Kv10.1-specific single-chain Fv antibodies fused to soluble tumor necrosis factor-related apoptosis-inducing ligand(scFv62-TRAIL), combined with the chemotherapeutic agent doxorubicin can overcome cancer cell resistance to chemotherapy and selectively induce apoptosis (98). lncRNA potassium voltage-gated channel subfamily Q member 1 overlapping transcript 1(KCNQ1OT1) is highly expressed in adriamycin-resistant AML cells, and induction of Tspan3 expression by adsorption of miR-193a-3p promotes the progression of adriamycin-resistant and drug-resistant AML cells (99).

KCa is a novel target for tumor prognosis as well as overcoming chemoresistance. Gemcitabine is the first-line treatment for metastatic BC. Increased expression of KCNN4 leads to resistance of BC cells to gemcitabine, which leads to cell proliferation and resistance to apoptosis and antimetabolites. This phenomenon is reversed *in vitro* and *in vivo* when KCNN4 is blocked using the specific inhibitor TRAM-34 or when KCNN4 is knocked down directly (100). Another study found that KCa3.1 channel activation has a significant impact on oncogenic Ca^2+^ signaling, DNA damage response, and radioresistance in the MMTV-PyMT BC model and can be used as a new target for radiation therapy and maintenance therapy (101). KCNN4 also plays a role in chemoresistance in CRC, and the KCa3.1 activator SKA-31 and Kv11.1 inhibitor E4031 have synergistic effects with cisplatin in causing apoptosis and inhibiting proliferation, helping to improve the efficacy of cisplatin and overcome cisplatin resistance in CRC (102). KCa3.1 channel blockers can increase the sensitivity of NSCLC cells to erlotinib and overcome resistance (103). Increased expression of KCa3.1 channels by radiation can suppress the pro-invasive phenotype induced by counteracting radiation in tumor cells and inhibit tumor resistance after radiation therapy in GBM patients (104).

Kir2.1 promotes cell growth by reducing drug-induced apoptosis and cell cycle arrest, resulting in multidrug resistance. Kir2.1 is involved in cell growth and chemoresistance by regulating MRP1/ABCC1 expression in small cell LC cell lines H69 and H446 cells and can be used as a prognostic factor for small cell LC as well as a new target for chemoresistance (105). These studies suggest that potassium channels are critical modulators of tumor resistance, and targeting potassium channels may be a promising therapeutic strategy to overcome tumor multidrug resistance.

### Role and mechanism of potassium channel in immunotherapy

**Figure 2:**
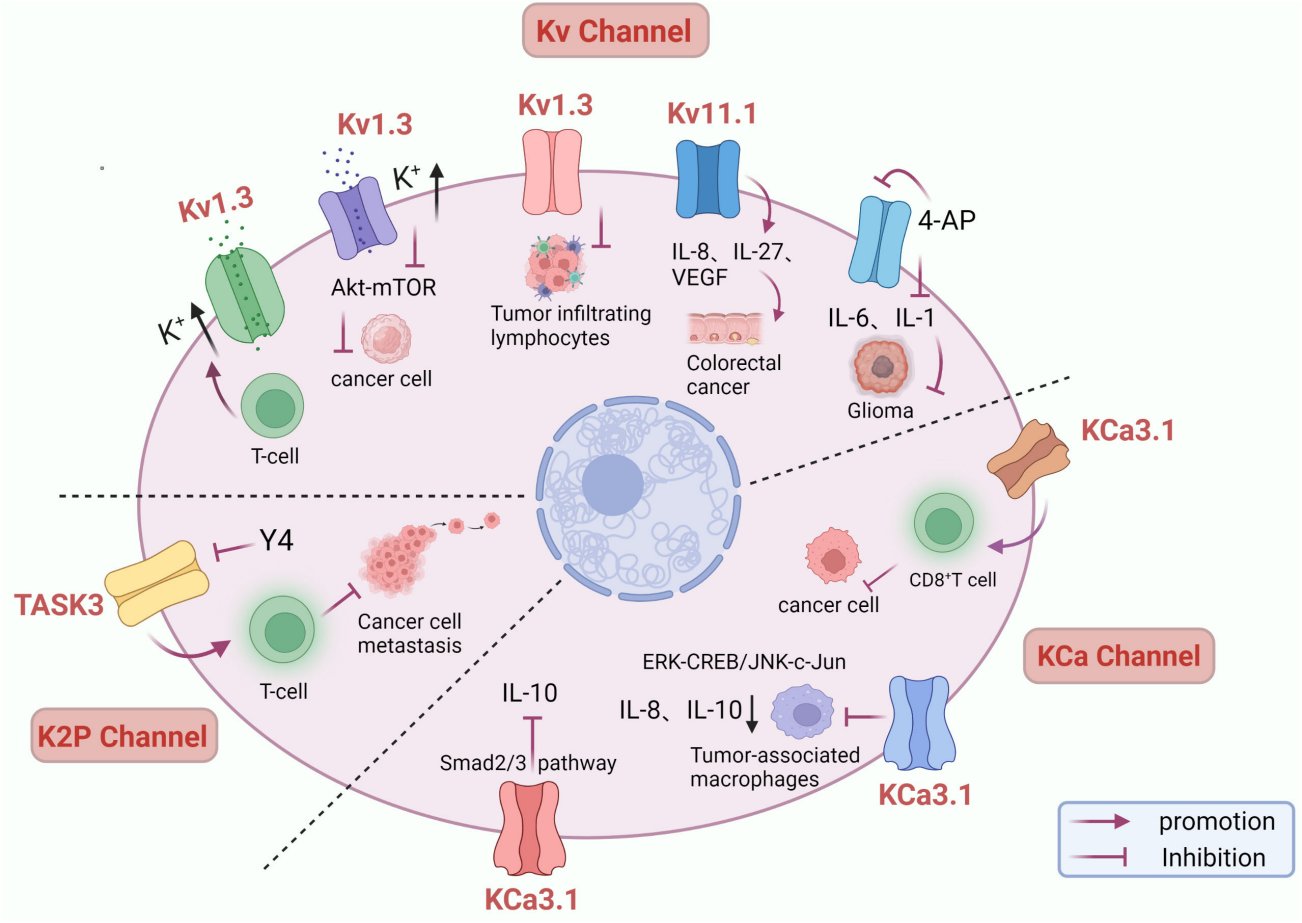
Role of potassium channels in immunotherapy. Inhibition of Kv1.3 channels leads to impaired tumor-infiltrating lymphocyte function and decreased ability to kill cancer cells. Kv1.3 overexpression decreases intracellular potassium and enhances antitumor function of T cells through activation of Akt – mTOR signaling. 4-AP blocked potassium channels and inhibited IL-6 and IL-1 secretion, inhibiting glioma cell proliferation. Up-regulation of Kv11.1 channel expression was accompanied by increased IL-8, IL-27, and VEGF, promoting colorectal cancer development. KCa3.1 activator suppress IL-10-induced tumor immune surveillance escape and IL-8-induced tumorigenicity and metastasis by inhibiting their production from TAMs through ERK-CREB and JNK-c-Jun cascades. KCa3.1 activator enhanced the killing effect of CD8 + T on tumor cells and induced IL-10 transcriptional inhibition in T lymphocytic leukemia through the Smad2/3 signaling pathway. Y4 mAb increases cell death by targeting inhibition of KCNK9 channels and activating anti-tumor immune responses.

Increasing evidence suggests that the immune microenvironment plays an essential role in tumor development and immunotherapy. In recent years, the study of potassium channels and cancer immunotherapy has gradually received attention (106). A study revealed a link between Kv1.3 channels and tumor infiltrating lymphocytes(TILs). A clinical study of head and neck cancer(HNC) found a 70% reduction in Kv1.3 channels in TILs, implying that loss of function of Kv1.3 channels in TILs may contribute to reduced immune surveillance in HNC (107). Kv1.3 is also a potential promoter of apoptosis. Intratumoral cell death increases extracellular K^+^ concentration, and high intracellular K^+^ inhibits T cell effector function (108). The expression level of the Kv11.1 channel is closely related to IL-8, IL-27 or VEGF, and the development of(CRC) also accompanies the up-regulation of the Kv11.1a expression level (109). Kv channel blocker 4-aminopyridine(4-AP) can inhibit the secretion of IL-6 and IL-1, thereby inhibiting glioma cell proliferation (110). Elevated extracellular potassium concentrations have been shown to inhibit T cell receptor(TCR) -driven Akt – mTOR phosphorylation and effector programs *in vitro*. Overexpression of the potassium channel Kv1.3 increases potassium efflux from tumor-specific T cells, thereby decreasing intracellular potassium ions, enhancing tumor clearance and survival in mice carrying melanoma, reducing tumor growth, and making T cells more effective in anticancer effects (111). The researchers found that KCa3.1 activators can enhance the killing effect on tumor cells by eliminating the inhibitory effect of adenosine on CD8^+^ T cell chemotaxis and promoting CD8^+^ T cell penetration into tumors (112). In CD8^+^ T cells, K^+^ efflux mediating KCNA3 activation increases antitumor function by promoting the expression of IFN-γ (111). KCa3.1 activators were able to significantly decrease the expression and secretion of the tumorigenic factors IL-8 and IL-10 in human monocytic leukemia derived M2 macrophages, and KCa3.1 activators inhibited IL-10 in tumor-associated macrophages(TAM) by ERK-CREB and JNK-c-Jun, thereby inhibiting IL-10-induced escape from tumor immune surveillance (113). Another study found that KCa3.1 activators have an inhibitory effect on IL-10 expression, and the Smad2/3 signaling pathway is involved in transcriptional inhibition of IL-10 in KCa3.1 activator-induced T-lymphoblastic leukemia HuT-78 cells, suggesting that KCa3.1 activators are a new therapeutic option that can inhibit IL-10 and evade the tumor-promoting activity of cancer immune surveillance (114). In addition, KCNN4 is significantly upregulated in many types of cancer tissues and plays an immunoregulatory role in the tumor microenvironment(TME), which is a powerful indicator of pan-cancer prognosis as well as immunotherapy (115). Extensive studies of KCa3.1 in immunotherapy have opened new avenues for treating tumors. Y4, as an antibody with the highest affinity binding, can induce channel internalization and inhibit the function of KCNK9, activate the anti-tumor immune response, increase cell death, and effectively inhibit the growth of human LC xenografts and mouse BC metastasis (116). Therefore, potassium channels play an important role in immunotherapy and may be an effective way to improve cancer immune surveillance and immunotherapy response (**Figure 2**).

## Conclusions and outlook

Cancer is a significant public health problem worldwide and is currently the leading cause of human death in the world, and its morbidity and mortality are also increasing year by year (117). The main treatment methods include surgery, chemotherapy, radiotherapy, and biological therapy. Continued development of new therapeutic modalities and drug targets is essential in cancer studies. Among the genes affected during oncogene transformation, genes encoding ion channels inevitably exist and play an essential role in cell proliferation, apoptosis, and neovascularization (4). As novel biomarkers of many cancers, potassium channels play an essential role in diagnosing, prognosis, and treating tumors. Among them, Kv10.1 has been extensively studied, and its overexpression is associated with poor prognosis in various cancers and can be used as a potential indicator and treatment strategy to predict chemosensitivity (96). High expression of genes encoding Kv11.1 channels is associated with favorable prognosis in estrogen receptor-negative breast cancer (75). Extensive expression of Kv11.1 channels in gastrointestinal tumors may serve as an early diagnostic marker (76). Expression of KCNN4 is associated with disease-free survival, immune infiltration, and several other clinicopathological features and can serve as a diagnostic and prognostic biomarker in TC (86). Abnormal expression of the KCNK gene family in BC can be used as an ideal prognostic biomarker for BC patients (118). It is also a powerful indicator of pan-cancer prognosis (115). In addition, drugs related to potassium channels have gradually become the focus of current research. The specific Kv1.3 channel inhibitor clofazimine can inhibit cancer cell proliferation and induce cancer cell apoptosis through the mitochondrial pathway (9). Potassium channel blockers can inhibit cell migration of human endometrial carcinoma and inhibit proliferation and induce apoptosis of rat glioma cells (3, 119). Kv11.1 activator NS1643 exerts antitumor effects in TNBC by causing DNA damage *via* a Ca^2+^ -dependent mechanism (74). KCa3.1 activators SKA-31 and Kv11.1 inhibitors can overcome cisplatin resistance in CRC and contribute to improving cisplatin efficacy (102). Therefore, potassium channels play a crucial role in the clinical treatment of cancer. However, the interaction mechanism of the combination has not been widely studied and can be used as the focus of further in-depth study.

In addition, potassium ions also play an important role in immunotherapy, and potassium ions are the key to controlling the anticancer ability of T cells (120). Studies have shown a positive association between serum potassium and cancer risk. For every 1 SD increase in potassium, the risk of overall cancer increased by 16%. Higher levels of serum potassium may contribute to cancer development and growth through immune mechanisms (121). Kv channel blocker 4-AP can inhibit the secretion of IL-6 and IL-1, thereby inhibiting glioma cell proliferation (110). KCa3.1 activators can enhance the killing effect of CD8 + T on tumor cells by eliminating the inhibitory effect of adenosine on CD8^+^ T cell chemotaxis (112). KCa3.1 activators have an inhibitory effect on IL-10 expression and can address the tumor-promoting activity of cancer immune surveillance by inhibiting IL-10 (114). Therefore, potassium channels may be an effective way to improve cancer immune surveillance and immunotherapy response.

Potassium channels have been intensively studied in the context of cancer. It is not only a biological marker of cancer but also an effective prognostic tool and provides a direction for targeted therapy and immunotherapy of cancer. Although ion channels hold great promise in targeted cancer therapy, they still face significant challenges, of which personalized therapy, drug treatment sensitivity, and off-target toxicity of targeted potassium channel drugs are still poorly studied (2). Therefore, it is necessary to improve the mechanism of potassium channels as drug targets and play the role of potassium channels in precision medicine.

## Author contributions

ML was responsible for collating and writing. PT, QZ and XM were responsible for collecting the data, and YZ was responsible for reviewing and proofreading. All authors contributed to the article and approved the submitted version.
